# Use of Tumor Necrosis Factor-α Antagonists Is Associated With Attenuated IgG Antibody Response Against SARS-CoV-2 in Vaccinated Patients With Inflammatory Bowel Disease

**DOI:** 10.3389/fimmu.2022.920333

**Published:** 2022-07-05

**Authors:** Antonius T. Otten, Arno R. Bourgonje, Petra P. Horinga, Hedwig H. van der Meulen, Eleonora A. M. Festen, Hendrik M. van Dullemen, Rinse K. Weersma, Coretta C. van Leer-Buter, Gerard Dijkstra, Marijn C. Visschedijk

**Affiliations:** ^1^ Department of Gastroenterology and Hepatology, University of Groningen, University Medical Center Groningen, Groningen, Netherlands; ^2^ Department of Medical Microbiology, University of Groningen, University Medical Center Groningen, Groningen, Netherlands

**Keywords:** inflammatory bowel disease, COVID-19, SARS-CoV-2, antibody, vaccination, TNF-α-antagonists

## Abstract

**Introduction:**

Patients with Inflammatory Bowel Disease (IBD) frequently receive immunomodulating treatment, which may render them at increased risk of an attenuated immune response upon vaccination. In this study, we assessed the effects of different types of commonly prescribed immunosuppressive medications on the serological response after vaccination against SARS-CoV-2 in patients with IBD.

**Methods:**

In this prospective observational cohort study, IgG antibody titers against SARS-CoV-2 were measured 2-10 weeks after completion of standard vaccination regimens in patients with IBD. Clinical characteristics, previous history of SARS-CoV-2 infection, type of vaccine (mRNA- or vector-based) and medication use were recorded at the time of sampling. Subsequently, a chemiluminescent microparticle immunoassay was used for the quantitative determination of IgG antibodies against the receptor-binding domain (RBD) of the S1 subunit of the spike protein of SARS-CoV-2.

**Results:**

Three hundred and twelve (312) patients with IBD were included (172 Crohn’s disease [CD] and 140 ulcerative colitis [UC]). Seroconversion (defined as titer of >50 AU/ml) was achieved in 98.3% of patients. Antibody concentrations were significantly lower in patients treated with TNF-α-antagonists *vs*. non-users of TNF-α-antagonists (geometric mean [95% confidence interval]: 2204 [1655-2935] *vs*. 5002 [4089-6116] AU/ml, *P*<0.001). In multivariable models, use of TNF-α-antagonists (*P*<0.001), vector vaccines (*P*<0.001), age (>50 years) (*P*<0.01) and CD (*P*<0.05) were independently associated with lower anti-SARS-CoV-2 antibody titers. In patients who received mRNA vaccines, users of thiopurines (either prescribed as monotherapy or in combination with biologicals) demonstrated significantly lower antibody titers compared to thiopurine non-users (*P*<0.05).

**Conclusion:**

Despite reassuring findings that most patients with IBD have detectable antibodies after anti-SARS-CoV-2 vaccination, TNF-α-antagonists were found to be strongly associated with an attenuated IgG antibody response after vaccination against SARS-CoV-2, independent of vaccine type, the time elapsed after vaccination and blood sampling, prior SARS-CoV-2 infection and patient age. Patients treated with thiopurines and receiving mRNA-based vaccines demonstrated lower anti-SARS-CoV-2 antibody titers compared with non-users.

## Introduction

The rapid onset of coronavirus disease 2019 (COVID-19, caused by the severe acute respiratory syndrome coronavirus-2, SARS-CoV-2) pandemic has resulted in a global health crisis (1). Extensive efforts have been made to control SARS-CoV-2 transmission and COVID-19 severity, primarily through the widespread implementation of vaccination campaigns to combat the spread of SARS-CoV-2. Patients with Inflammatory Bowel Disease (IBD) were excluded from the first human trials investigating the efficacy, safety and tolerability of anti-SARS-CoV-2 vaccines. IBD expert consensus recommendations state that the protective properties of vaccination outweigh potential disadvantages, thus actively encouraging patients to pursue vaccination (2, 3). However, the lack of real-world data from well-structured, prospective observational cohort studies raises important concerns for this specific patient population as well as several questions that remain unanswered (4).

In this respect, the variety of prescribed anti-inflammatory and immunomodulatory drugs to induce and maintain disease remission in patients with Crohn’s disease (CD) and ulcerative colitis (UC) may influence an individual’s susceptibility to contract COVID-19 as well as the ability to mount an adequate serological response upon vaccination against SARS-CoV-2. Medical treatment for IBD aims to suppress disease activity, but concurrently compromises the immune system and its ability to respond to invading pathogens. Patients with IBD are thus at increased risk of opportunistic and viral infections when treated with immunosuppressive agents (5, 6). Considering that IBD affects a substantial proportion of the worldwide population, especially in Westernized countries (7), it is evident that serological responses after vaccination against SARS-CoV-2 deserve further attention.

Reassuringly, current data do not indicate the existence of an increased susceptibility to contract SARS-CoV-2 in patients with IBD receiving immunosuppressive treatment, nor do patients with IBD experience a more severe disease course after SARS-CoV-2 infection (8, 9). However, early findings suggest that certain immunosuppressive agents, especially TNF-α-antagonists, may have attenuating effects on the serological response after SARS-CoV-2 infection (10, 11). Immunosuppressive therapy is known to impair the seroconversion rates after vaccination against influenza, viral hepatitis and pneumococcus (4). These observations lead to concerns regarding a potentially attenuated serological response after vaccination against SARS-CoV-2.

In this study, we aimed to assess the effects of different types of immunosuppressive medications on the serological response after vaccination against SARS-CoV-2 in patients with IBD in a prospective, real-world setting. Additionally, we aimed to identify demographic and clinical risk factors associated with a possibly attenuated immune response against SARS-CoV-2 vaccination.

## Materials and Methods

### Study Design and Study Population

This study was a prospective observational cohort study. Patients were included from the IBD center of the University Medical Center Groningen (UMCG), the Netherlands, between May 2021 and October 2021. Patients received a personal invitation by mail to supply a blood sample after they completed a standard vaccination regimen against SARS-CoV-2. IgG antibody titers against the spike receptor-binding domain (RBD) of SARS-CoV-2 were determined in serum. Inclusion criteria were an established diagnosis of CD or UC through the standard endoscopic, histological, and radiological criteria, an age of 18 years or older, and full vaccination against SARS-CoV-2. Full vaccination was defined as a completion of a vaccination regimen in accordance with practice guidelines, which constitutes either a double vaccination of Pfizer–BioNTech BNT162b2, Moderna mRNA-1273 or AstraZeneca AZD1222 vaccine, a single vaccination of Janssen Ad26.CoV2.S vaccine, or a previous confirmed SARS-CoV-2 infection followed by a single vaccination.

### Data Collection

Detailed phenotypic and demographic data were collected for all patients, including age, sex, body-mass index (BMI), smoking status, Montreal disease classification, medication use, history of bowel surgery, disease activity, and comorbidities, all of which were assessed at the time of antibody measurements. Standard laboratory parameters were recorded if available and within a span of 3 months at the time of sampling. Patients were asked to complete a survey detailing previous history and date of SARS-CoV-2 infection, type of vaccine, and date of last vaccination.

### Study Outcomes

The primary study outcome was the anti-SARS-CoV-2 spike (S) antibody concentration 2-10 weeks after full vaccination. Secondary outcome measures were the rates of seroconversion (defined as a response of >50 AU/ml) and loss of SARS-CoV-2 antibody response over time.

### Serological Detection of Anti-SARS-CoV-2 Antibodies

Analysis was performed at the microbiological laboratory of the University Medical Center Groningen, the Netherlands. The SARS-CoV-2 IgG II Quant ELISA method (Architect, Abbott, IL, USA), a chemiluminescent microparticle immunoassay, was used for the quantitative determination of IgG antibodies against the spike receptor-binding domain (RBD) of the S1 subunit of the spike protein of SARS-CoV-2 (12). Serum and plasma samples were tested according to the manufacturer’s instructions. Results were expressed in AU/ml, with 50 AU/mL as a positive cut-off and a maximal threshold of quantification of 40,000 AU/ml. Calibration with the WHO reference serum for antibody detection showed that 50 AU/ml corresponded to 7 BAU/ml.

### Statistical Analysis

Baseline demographic and clinical characteristics of the study population were presented as means ± standard deviations (SDs), median with interquartile ranges (IQR), or proportions with corresponding percentages (%). Normality assessment was performed by visual inspection of normal probability (Q-Q) plots and histograms and confirmed using Shapiro-Wilk tests. Anti-SARS-CoV-2 antibody concentrations were log-transformed before entry into analysis. Data were presented as geometric means (geometric SD) of log-transformed antibody titers. Univariable associations between demographic and clinical characteristics and log-transformed anti-SARS-CoV-2 antibody titers were computed using Spearman’s rank correlation coefficients for continuously distributed variables and independent sample *t*-tests were used for categorical variables. Multivariable linear regression analyses (method: enter) were conducted using log-transformed anti-SARS-CoV-2 antibody titers as the main outcome variable. Multivariable analyses were performed to identify independently associated factors with SARS-CoV-2 seropositivity, while *a priori* adjusting for the effects of patient age, prior SARS-CoV-2 infection, and the time interval between last vaccination and sampling, and adjusting for multiple comparisons under a false discovery rate (FDR) of 5%. Data were analyzed using SPSS Statistics 25.0 software package (IBM Corp.) and the Python programming language (v.3.8.5, Python Software Foundation, https://www.python.org) using the *pandas* (v.1.2.3) and *numpy* (v.1.20.0) packages. Data visualization was performed using the *seaborn* (v.0.11.1) and *matplotlib* (v.3.4.1) packages in Python. *P*-values <0.05 were considered statistically significant.

### Ethical Considerations

All patients provided written informed consent for the use of patient data and serum by consenting to participate in the Dutch Parelsnoer IBD Biobank (13). The study was conducted according to the principles of the Declaration of Helsinki (2013).

## Results

### Cohort Characteristics

**Figure 1:**
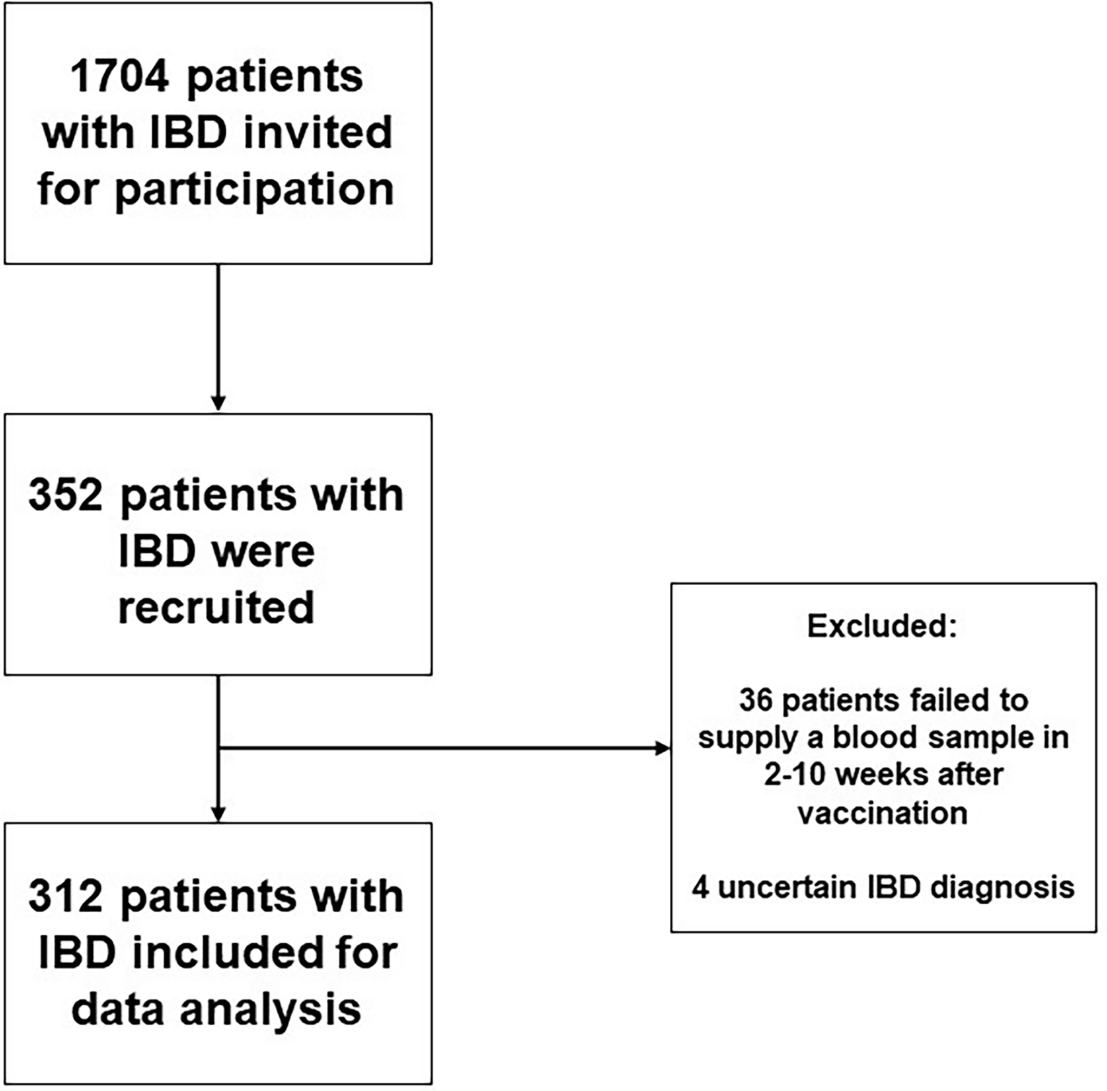
Flow chart of the study showing the participant inclusion procedure.

Initially, 352 patients with IBD were recruited, of which 40 patients were excluded because no serum sample was collected within 2-10 weeks after administration of last vaccination (n=36) or because of an uncertain diagnosis of IBD (n=4) (**Figure 1**)

In total, 312 patients were included for analysis, of which 172 were diagnosed with CD and 140 diagnosed with UC. Overall, more females had CD, while more men had UC (P<0.05). The median age at time of sampling was 49 [39;59] years in patients with CD compared with 52 [38;63] years in patients with UC (P=0.33). Concerning the use of biologics, patients with CD were more often treated with TNF-α-antagonists (n=79, 45.9%, i.e., infliximab, adalimumab, certolizumab and golimumab) and ustekinumab (n=15, 8.7%) compared to patients with UC (TNF-α-antagonists, n=26 (18.6%); ustekinumab: n=1, (0.7%)) P<0.01 for both). Patients with UC were more frequently treated with vedolizumab (n=15, 10.7%) compared with CD (n=4, 2.3%; P<0.01). Patients with CD more often underwent IBD-related surgical interventions compared with patients with UC (P<0.01). In total, 15 (4.8%) patients had a history of a PCR-test confirmed SARS-CoV-2 infection. All demographic and clinical characteristics of the study population are presented in **Table 1**.

**Table 1:** Demographic and clinical characteristics of the study population.

Variable	CD	UC	*P*-value
	*n* = 172	*n* = 140	
Anti-SARS-CoV-2 antibody titer (AU/mL)	3945 [1463;10115]	5067 [2074;12906]	0.10
Sex, *n* (%)			0.05
*Male*	55 (32.0)	60 (42.9)	
*Female*	17 (68.0)	80 (57.1)	
Age (years)	49 [36;59]	52 [38;63]	0.33
Current smoking, *n* (%)			0.52
*Yes*	22 (13.8)	14 (11.3)	
*No*	137 (86.2)	110 (88.7)	
BMI, kg/m^2^	24.6 [21.9;27.4]	25.3 [22.6;29.7]	<0.05
Montreal classification			
Montreal Age (A)			0.15
A1 (≤ 16 years)	22 (12.8)	14 (10.0)	
A2 (17-40 years)	111 (64.5)	72 (51.4)	
A3 (> 40 years)	39 (22.7)	42 (30.0)	
Montreal Location (L)			
L1 (ileal disease)	41 (23.8)	–	
L2 (colonic disease)	35 (20.3)	–	
L3 (ileocolonic disease)	76 (44.2)	–	
L4 (upper GI disease)	19 (11.0)	–	
L1 + L4	4 (2.3)	–	
L2 + L4	4 (2.3)	–	
L3 + L4	11 (6.4)	–	
Montreal Behavior (B)			
B1 (nonstricturing, nonpenetrating)	69 (40.1)	–	
B2 (stricturing)	30 (17.4)	–	
B3 (penetrating)	14 (8.1)	–	
B1 + P (perianal disease)	23 (13.4)	–	
B2 + P (perianal disease)	24 (14.0)	–	
B3 + P (perianal disease)	11 (6.4)	–	
Montreal Extension (E)			
E1 (proctitis)	–	16 (11.4)	
E2 (left-sided colitis)	–	37 (26.4)	
E3 (pancolitis)	–	67 (47.9)	
Medication use, *n* (%)			
Aminosalicylates	10 (5.8)	95 (67.9)	<0.01
Thiopurines	72 (41.9)	45 (32.1)	0.08
Steroids	30 (17.4)	14 (10.0)	0.06
Methotrexate	5 (2.9)	2 (1.4)	0.47
TNF-α-antagonists^^^	79 (45.9)	26 (18.6)	<0.01
Vedolizumab	4 (2.3)	15 (10.7)	<0.01
Ustekinumab	15 (8.7)	1 (0.7)	<0.01
Disease activity			
HBI score (CD)	109 (63.4)	–	
Remission < 5	89 (81.7)	–	
Mild disease 5-7	12 (11.0)	–	
Moderate disease 8-16	8 (7.3)	–	
Severe disease >16	0 (0.0)	–	
SCCAI score (UC)	–	62 (44.3)	
Remission ≤ 2	–	53 (85.5)	
Active disease > 2	–	9 (14.5)	
CRP (mg/L)	1.6 [0.7-5.0]	1.9 [0.8-3.8]	0.86
FCP (μg/g)	128 [45-361]	120 [26-695]	0.95
Surgical history, *n* (%)	81 (47.1)	16 (11.4)	<0.01

Data are presented as median [IQR] or proportions n with corresponding percentages (%). Abbreviations: CD, Crohn’s disease; BMI, body-mass index; CRP, C-reactive protein; FCP, fecal calprotectin; HBI, Harvey-Bradshaw Index; SCCAI, Simple Clinical Colitis Activity Index; UC, ulcerative colitis.

### Univariate Associations Between Patient Characteristics and Anti-SARS-CoV-2 Antibody Titers

**Figure 2:**
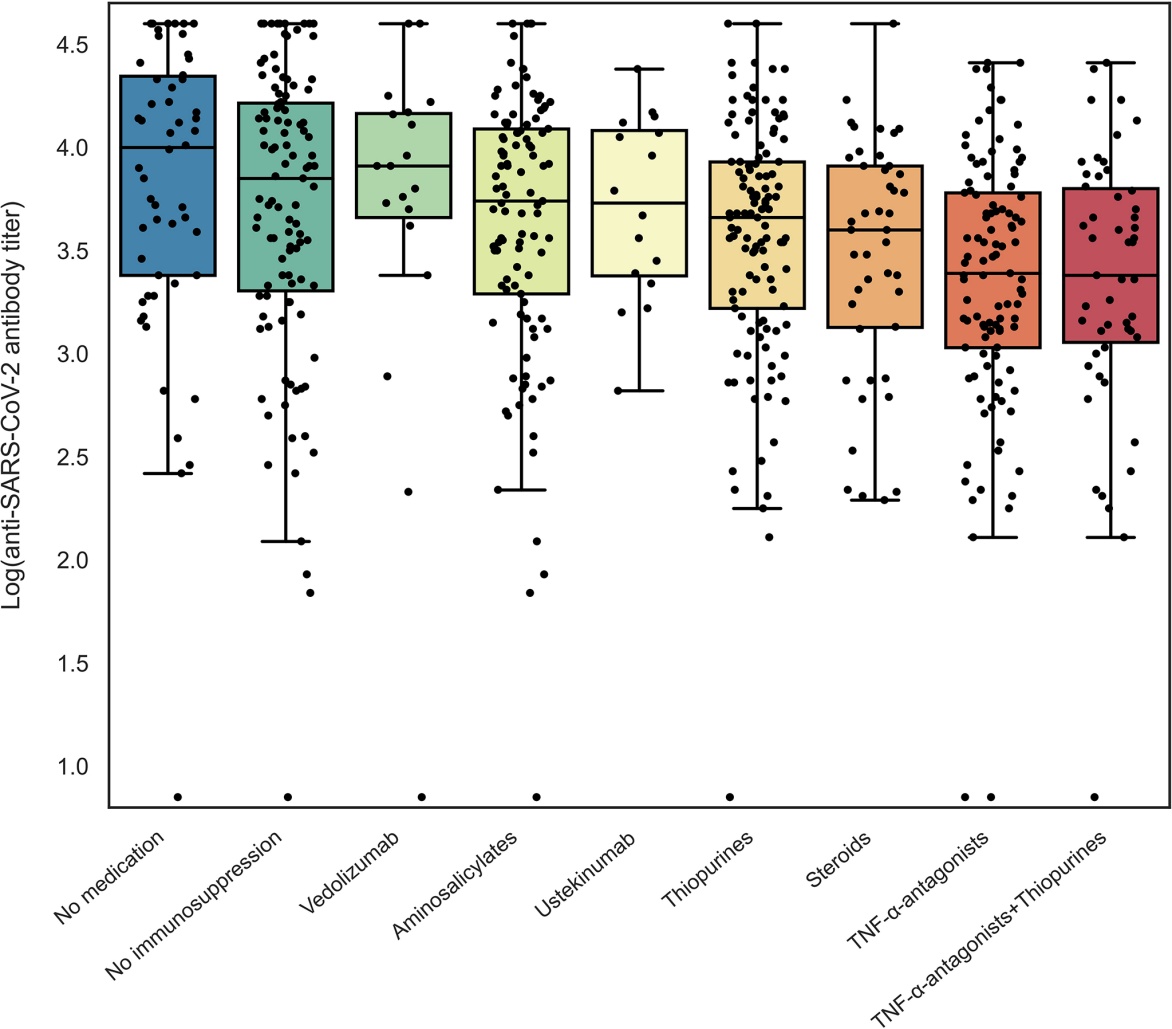
Log-transformed anti-SARS-CoV-2 antibody titers for patients with IBD according to different types of medications used. Patients using a specific type of medication were compared to non-users for log-transformed anti-SARS-CoV-2 antibody titers using independent sample t-tests (see **Table 2**).

**Figure 3:**
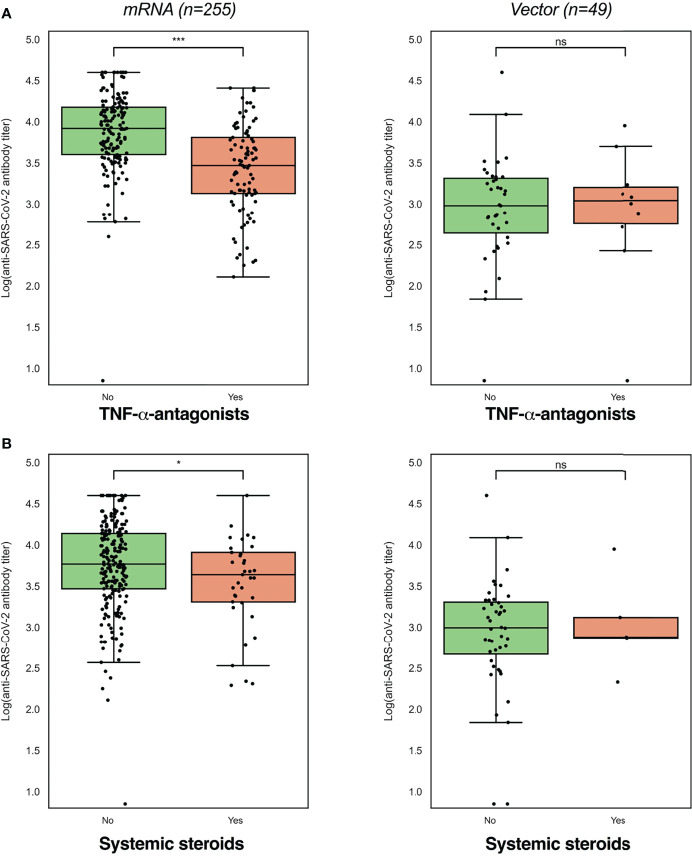
Log-transformed anti-SARS-CoV-2 antibody titers were lowest in patients using TNF-α-antagonists **(A)** and systemic steroids (**B**), while these differences were dependent on the vaccine type received (mRNA or vector-type). Log-transformed anti-SARS-CoV-2 antibody titers were compared between users and non-users with independent sample *t*-tests (see **Table 2**). ^*^P<0.05; ^***^P<0.001.

Seroconversion was achieved in 98.3% of patients. Anti-SARS-CoV-2 antibody concentrations were not normally distributed and positively skewed (Shapiro-Wilk test, P<0.001), and presented as log-transformed antibody titers. An overview of associations between demographic and clinical characteristics and log-transformed anti-SARS-CoV-2 antibody titers can be found in **Table 2**. In general, patients who had a history of COVID-19 and patients who were vaccinated with mRNA-based vaccines demonstrated significantly higher anti-SARS-CoV-2 antibody concentrations compared to the group of patients without a history of prior COVID-19 infection and vector-type vaccines, respectively (both P<0.001). Patient age (⍴=-0.191, P=0.001) and the time that had elapsed since the last vaccination (⍴=-0.146, P=0.01) were both significantly inversely associated with anti-SARS-CoV-2 antibody concentrations. Regarding medication use, patients who were treated with TNF-α-antagonists showed the lowest antibody titers compared to patients who were not treated with TNF-α-antagonists (geometric mean [95% confidence interval]: 2204 [1655-2935] *vs*. 5002 [4089-6116] AU/ml, P<0.001) (**Table 2**, **Figure 2**). This difference was particularly present in patients who were vaccinated with mRNA-based vaccines compared to vector-based vaccines (**Figure 3**). There was no difference in antibody titers when comparing patients who received monotherapy with TNF-α-antagonists *vs*. patients who were treated with TNF-α-antagonists in combination with immunomodulating drugs (**Figure 2**). Besides TNF-α-antagonists, patients who received mRNA vaccines and who were being treated with systemic steroids also demonstrated lower anti-SARS-CoV-2 antibody concentrations (geometric mean [95% confidence interval]: 2963 [1978-4437] *vs*. 3954 [3282-4763] AU/ml, P<0.05).

**Table 2:** Univariable associations between patient characteristics and log-transformed anti-SARS-CoV-2 antibody titers.

Variable	Categories	Total		mRNA vaccines		Vector vaccines^†^	
		*n = 312*		*n = 255*		*n = 49*	
		Value	*P*-value	Value	*P*-value	Value	*P*-value
**Demographics**							
Age (years)		-0.191	**0.001**	-0.098	0.120	0.074	0.611
Sex	Male	3.59 (0.63)	0.907	3.69 (0.58)	0.560	3.06 (0.64)	0.552
	Female	3.58 (0.68)		3.73 (0.54)		2.85 (0.69)	
Smoking	Never	3.65 (0.68)	0.112	3.75 (0.58)	0.390	2.76 (0.88)	0.505
	Former	3.57 (0.63)		3.70 (0.53)		3.06 (0.55)	
	Current	3.39 (0.76)		3.59 (0.59)		2.60 (0.93)	
BMI, kg/m^2^		-0.030	0.596	0.026	0.681	0.011	0.940
Time since last vaccination (days)		-0.146	**0.010**	-0.204	**0.001**	0.010	0.947
Prior SARS-CoV-2 infection	No	3.55 (0.66)	**0.001**	3.69 (0.55)	**0.002**	–	–
	Yes	4.15 (0.36)		4.17 (0.37)		–	
Type of vaccine	Vector	2.93 (0.68)	**<0.001**	–	**-**	–	–
	mRNA	3.71 (0.56)		–		–	
Diagnosis	CD	3.53 (0.67)	0.151	3.64 (0.55)	**0.016**	2.85 (0.82)	0.855
	UC	3.64 (0.64)		3.81 (0.55)		2.98 (0.56)	
**Medication use**							
Aminosalicylates	No	3.56 (0.66)	0.403	3.66 (0.55)	**0.021**	2.94 (0.84)	0.779
	Yes	3.62 (0.66)		3.83 (0.56)		2.92 (0.46)	
Thiopurines	No	3.59 (0.70)	0.616	3.75 (0.58)	0.182	2.82 (0.75)	0.101
	Yes	3.56 (0.59)		3.66 (0.51)		3.15 (0.44)	
Steroids	No	3.60 (0.67)	0.244	3.74 (0.55)	**0.032**	2.92 (0.69)	0.974
	Yes	3.47 (0.58)		3.53 (0.55)		3.03 (0.59)	
Methotrexate	No	3.59 (0.66)	0.050	3.73 (0.55)	**0.012**	-	-
	Yes	3.12 (0.64)		3.16 (0.69)		-	
TNF-α-antagonists	No	3.70 (0.64)	**<0.001**	3.88 (0.49)	**<0.001**	2.94 (0.64)	0.931
	Yes	3.34 (0.64)		3.42 (0.55)		2.90 (0.84)	
TNF-α-antagonists + Immunomodulators	No	3.39 (0.62)	0.468	3.47 (0.49)	0.473	2.51 (1.56)	0.569
	Yes	3.30 (0.66)		3.39 (0.60)		3.06 (0.38)	
Vedolizumab	No	3.57 (0.65)	0.485	3.71 (0.53)	0.378	2.93 (0.69)	0.835
	Yes	3.68 (0.87)		3.84 (0.88)		2.87 (0.52)	
Ustekinumab	No	3.57 (0.67)	0.423	3.71 (0.56)	0.837	-	-
	Yes	3.71 (0.44)		3.74 (0.44)		-	
**Disease activity**							
HBI score (CD)		-0.158	0.101	-0.061	0.556	-0.611	**0.046**
SCCAI (UC)		-0.011	0.933	-0.008	0.957	-0.137	0.613
**Surgical history**	No	3.59 (0.59)	0.663	3.70 (0.53)	0.666	2.93 (0.43)	0.571
	Yes	3.56 (0.80)		3.74 (0.61)		2.92 (1.01)	

Data are presented as mean (SD) of log-transformed anti-SARS-CoV-2 IgG antibody titers for categorical variables or as Spearman rank correlation coefficients (rho) in case of continuous variables, where P-values are derived from independent sample t-tests or Spearman’s rho, respectively. ^†^P-values for categorical variables calculated by non-parametric tests due to small and imbalanced group sizes. Bold P-values indicate statistical significance.

In addition, mRNA-vaccinated patients who were treated with thiopurines showed a trend towards lower antibody concentrations compared to patients who were not treated with thiopurines (geometric mean [95% confidence interval]: 4523 [3574-5724] *vs*. 5634 [4561-6958] AU/ml, P=0.182). Finally, patients who were treated with vedolizumab, ustekinumab, or aminosalicylates did not have lower anti-SARS-CoV-2 antibody concentrations compared to those who did not receive the respective medications.

### Multivariable Associations Between Patient Characteristics and Medication Use and Anti-SARS-CoV-2 Antibody Titers

**Figure 4:**
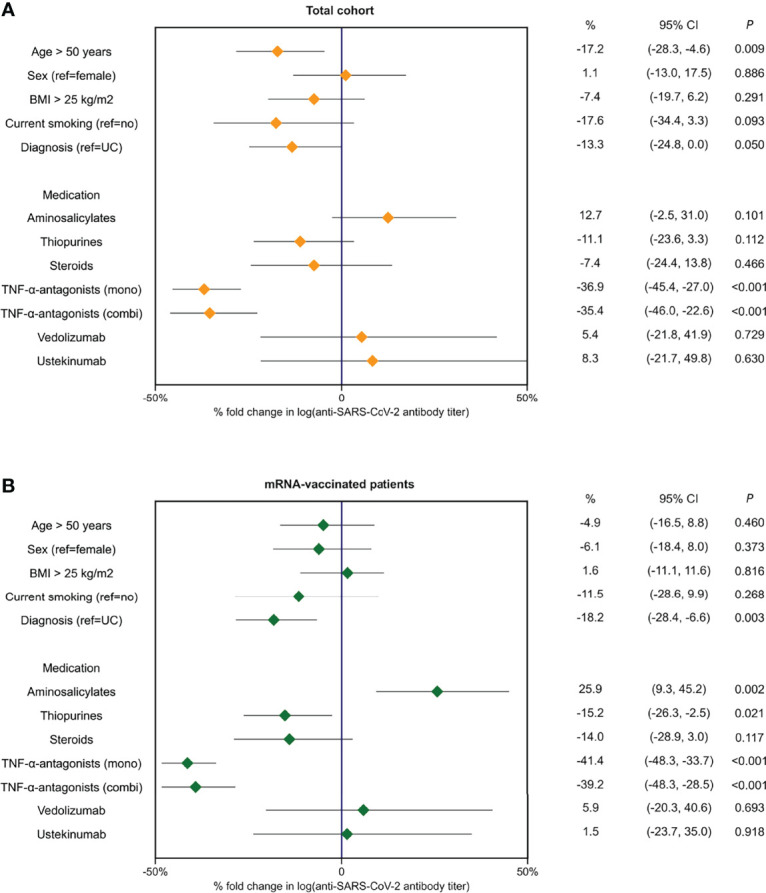
Forest plots demonstrating percentages (%) of fold change in log-transformed antibody titers based on exponentiated coefficients derived from multivariable linear regression analyses in the full cohort of patients with IBD **(A)** and in patients who received mRNA-type vaccines (**B**).

In multivariable linear regression analyses, with *a priori* adjustment for patient age, prior SARS-CoV-2 infection and the time elapsed since last vaccination was received, the use of TNF-α-antagonists (percentage decrease -37%, P<0.001), patient age (>50 years) (-17%, P<0.01), and the diagnosis of CD (*vs*. UC) (-13%, P<0.05) were associated with anti-SARS-CoV-2 antibody titers (**Table 3**, **Figure 4**). In patients who were vaccinated with mRNA-based vaccines, similar results were observed, although thiopurine use emerged as an additional factor that was negatively associated with anti-SARS-CoV-2 antibody concentrations, independently of factors that were controlled for (percentage decrease -15%, P<0.05). For this association, however, statistical significance vanished when patients using concomitant TNF-α-antagonists were excluded (i.e., only selecting patients on thiopurine monotherapy) (P=0.08). Finally, patients who received mRNA-vaccines and were treated with aminosalicylates demonstrated relatively higher antibody concentrations (percentage increase +26%, P<0.01) compared with non-users.

**Table 3:** Multivariable linear regression analysis showing non-exponentiated beta-coefficients for associations between patient characteristics and log-transformed anti-SARS-CoV-2 antibody titers in patients with IBD, for the total cohort and for mRNA vaccines separately.

Variable	Total^†^			mRNA vaccines		
	*n = 312*			*n = 255*		
	Beta	95% CI	*P*-value	Beta	95% CI	*P*-value
Age >50 years	-0.189	-0.332;-0.047	**0.009**	-0.050	-0.185;0.084	0.460
Sex (ref=female)	0.011	-0.139;0.161	0.886	-0.063	-0.203;0.077	0.373
Current smoking (ref=never/ever)	-0.194	-0.421;0.032	0.093	-0.122	-0.337;0.094	0.268
BMI > 25 kg/m^2^	-0.077	-0.220;0.066	0.291	0.016	-0.118;0.150	0.816
Diagnosis (ref=UC)	-0.143	-0.285;0.000	**0.050**	-0.201	-0.334;-0.069	**0.003**
Vaccine type (ref=vector)	0.735	0.554;0.915	**<0.001**	–	–	**-**
**Medication use**						
Aminosalicylates	0.127	-0.025;0.279	0.101	0.231	0.089;0.373	**0.002**
Thiopurines	-0.118	-0.269;0.032	0.112	-0.165	-0.305;-0.025	**0.021**
Steroids	-0.076	-0.282;0.129	0.466	-0.151	-0.341;0.038	0.117
TNF-α-antagonists	-0.460	-0.605;-0.315	**<0.001**	-0.535	-0.659;-0.411	**<0.001**
TNF-α-antagonists + Immunomodulators	-0.437	-0.617;-0.256	**<0.001**	-0.497	-0.659;-0.336	**<0.001**
Vedolizumab	0.053	-0.246;0.351	0.729	0.057	-0.227;0.341	0.693
Ustekinumab	0.080	-0.245;0.404	0.630	0.015	-0.270;0.300	0.918

**^†^** Adjusted for: prior SARS-CoV-2 infection, patient age, and time since last vaccination. Bold P-values indicate statistical significance.

### Anti-SARS-CoV-2 Antibody Concentrations Decrease Over Time

**Figure 5:**
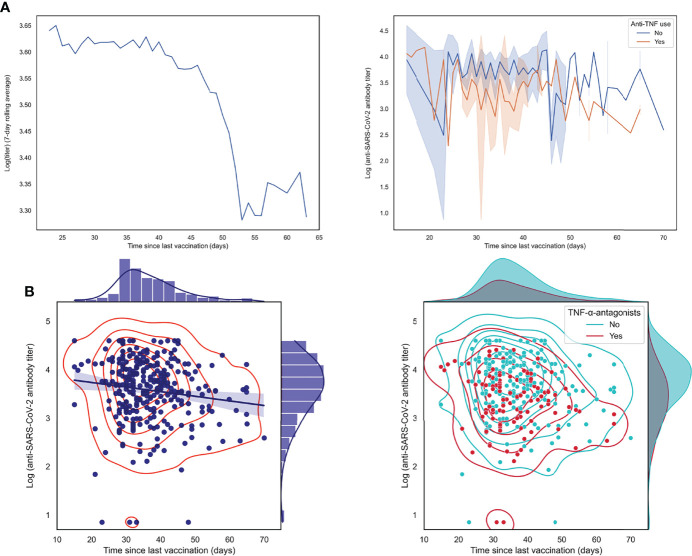
Log-transformed anti-SARS-CoV-2 antibody titers are negatively associated with the elapsed time since the last vaccination was received. **(A)** Patients with IBD who received their last vaccination less recently show lower antibody titers, as demonstrated by a 7-day rolling average and by individually connected data points stratified by use of TNF-α-antagonists. **(B)** Scatter plots with associated kernel density estimations showing an inverse association between anti-SARS-CoV-2 antibody titers (log-transformed) and time since last vaccination was received (left lower panel), an association which was not significantly modified by the use of TNF-α-antagonists (lower right panel).

Finally, we aimed to study the association between anti-SARS-CoV-2 antibody concentrations and the elapsed time since the last vaccination in more detail. When calculating a seven-day rolling average of log-transformed anti-SARS-CoV-2 antibody concentrations, we observed an evident drop in antibody concentrations after approximately six weeks after the last vaccination was received (**Figure 5**). Here, patients who were treated with TNF-α-antagonists demonstrated lower antibody concentrations but did not show a significantly faster decline in antibody concentrations. In line with this observation, there was no significant effect modification between the use of TNF-α-antagonists and elapsed time since last vaccination (P-value for interaction: 0.569) (**Figure 5B**).

## Discussion

In this prospective cohort consisting of patients with IBD, we investigated the influences of commonly described immunosuppressive agents on the serological response after full vaccination against SARS-CoV-2. We demonstrated that patients treated with TNF-α-antagonists presented with significantly lower anti-SARS-CoV-2 antibody concentrations, compared to TNF-α-antagonists non-users. In patients who received mRNA vaccines, treatment with thiopurines emerged as an additional independent factor that was associated with reduced antibody concentrations. In addition, patients with CD had lower anti-SARS-CoV-2 antibody concentrations compared to patients with UC, and patient age was inversely associated with anti-SARS-CoV-2 antibody concentrations. Finally, we observed a negative association between the elapsed time since last vaccination and antibody concentrations. Taken together, our results suggest that the use of TNF-α-antagonists is clearly associated with attenuated IgG antibody response against SARS-CoV-2 in fully vaccinated patients with IBD, while the use of thiopurines may also be potentially associated with an attenuated serological response.

Direct data comparisons with other cohorts are obscured due to differences in the type of antibody detection assay that was used, yet our primary findings are well in line with other observational studies investigating serological response after vaccination against SARS-CoV-2 in patients with IBD (14). In our study, 98.3% of patients showed detectable SARS-CoV-2-IgG antibodies 2-10 weeks after their last vaccination, which is comparable to previously reported seroconversion rates (15, 16). The finding that impairment of SARS-CoV-2-IgG response is most pronounced in TNF-α-antagonist-treated patients is also supported by distinctive cohorts (14, 17–19). The CLARITY-IBD study, consisting of a proportionate observational cohort of patients with IBD, reported considerably lower seroconversion rates after first vaccination in infliximab-treated patients compared to vedolizumab-treated patients, while the administration of the second dose led to comparable rates of 85% seroconversion (17). In our study, we found detectable SARS-CoV-2 antibodies in 96.9% of patients treated with TNF-α-antagonists with no prior PCR-test-confirmed SARS-CoV-2 infection. These seroconversion rates in immunosuppressed patients with IBD are supported by similar recent cohorts (20, 21).

Despite these convincing signs of a humoral response after full vaccination, certain aspects demand careful consideration. For example, a recently published study reported a five-fold reduction in anti-SARS-CoV-2 antibody concentrations among patients receiving TNF-α-antagonists, which was a rapid decay when compared with patients who were treated with vedolizumab, who showed sustained antibody concentrations 16 weeks after the last vaccination (22). Besides this accelerated reduction in neutralizing antibodies in infliximab-treated patients, neutralizing antibody-induced effects were additionally found to be reduced in TNF-α-antagonists users (18).

Efficient TNF-α neutralization may play an important role in the diminished serological response after vaccination (11). However, contradictory findings exist whether TNF-α-antagonist drug levels affect serological response after vaccination against SARS-CoV-2 (11, 18). Alterations in serological response in patients with IBD treated with TNF-α-antagonists may be explained through analysis of the affected pathways. Neutralization of TNF-α is known to disrupt the T-cell dependent humoral immune response, which leads to a reduction in B-cell maturation (23, 24).

Besides TNF-α-antagonists, we also observed that thiopurine use was significantly associated with reduced IgG antibody response against SARS-CoV-2 in patients who received mRNA-based vaccines. However, the concomitant use of thiopurines and TNF-α-antagonists did not demonstrate further reduced antibody responses compared with TNF-α-antagonist monotherapy. Thiopurines, including the use of azathioprine, mercaptopurine and thioguanine, are commonly used immunomodulators in IBD and have been variably associated with reduced vaccine responses (17, 19) For example, the CLARITY-IBD study demonstrated an association between immunomodulator use and lower seroconversion rates following both the first and second dose of vaccination, as well as reduced anti-SARS-CoV-2 antibody concentrations when patients used immunomodulators alongside TNF-α-antagonists (17, 25). Likewise, a previous study conducted in solid organ transplant recipients showed reduced antibody formation in patients who were using antimetabolite agents including azathioprine and mycophenolate mofetil (26). However, another study did not demonstrate reduced antibody formation against SARS-CoV-2 for any type of IBD medication except the use of systemic steroids, for which we only observed reduced antibody concentrations in univariate analysis (9). Additionally, the recent VIP-study identified tofacitinib as an immunosuppressant with a substantial immunogenicity-attenuating effect (27). Although the serological response (i.e., the degree of antibody formation) against SARS-CoV-2 vaccinations, as well as against other types of vaccinations such as influenza, pneumococcal and hepatitis B vaccines (4) may be attenuated; the overall serological response is generally deemed sufficient.

In the present study, we identified older age, having a diagnosis of CD instead of UC, and having received a mRNA-based vaccine (*vs*. vector-based vaccines) as main risk factors for reduced anti-SARS-CoV-2 antibody concentrations. These findings corroborate those from previous reports, especially those from the CLARITY-IBD study, showing older age, immunomodulator use, CD diagnosis (*vs*. UC or IBD-unclassified), and current smoking as main risk factors (17, 25). In addition, we observed a trend towards reduced antibody concentrations in current smokers, which has also been found previously as well as in relation to other types of vaccinations, e.g., against influenza and hepatitis B (28, 29). Although patients with CD tended to have lower antibody concentrations compared with patients with UC, previous vaccination studies have not demonstrated attenuated immunogenicity against influenza (4) or pneumococcal polysaccharide (30) vaccinations that are specific to patients with CD, even in the absence of immunosuppressive treatment. Finally, we also identified comparatively higher anti-SARS-CoV-2 antibody concentrations in patients who received mRNA-based vaccines *vs*. adenoviral vector-based vaccines. Although both groups were highly imbalanced in our study (mRNA, n=255 *vs*. vector-based vaccines, n=49, an approximate ratio of 5:1), these differences in IgG antibody concentrations favoring mRNA vaccines suggests that it is important to acknowledge potential differential patterns of immunogenicity and S1-specific IgG kinetic profiles attributed to different vaccine platforms. Indeed, recent studies indicate that individuals receiving mRNA-based vaccines generally mount higher and faster antibody responses compared with individuals receiving vector-based vaccines (20, 31–33).

Despite an apparent attenuated serological response after full vaccination in patients with IBD who were treated with TNF-α-antagonists and/or thiopurines, almost all patients (98.3%) demonstrated seroconversion. However, recent findings from the CLARITY-IBD cohort report an accelerated antibody decay and an increased risk for SARS-CoV-2 breakthrough infections after vaccination when comparing infliximab treated patients to vedolizumab treated patients. This enhanced risk was found to be associated with peak IgG antibody concentrations after second vaccination dose (22). It should be carefully investigated whether patients with IBD receiving immunosuppressive therapy also show a stronger reduction in antibody concentrations over a longer follow-up duration, whether these reductions will lead to growing hospitalization rates and if these reductions can be combated by booster vaccinations (22, 34, 35). Furthermore, the international SECURE-IBD registry is also of particular value as it collects information on the disease course in patients with IBD who have been infected and vaccinated with SARS-CoV-2. For instance, the use of systemic steroids was identified as a risk factor for a more severe disease course and hospitalization. A combination of thiopurines with TNF-α-antagonists as compared to TNF-α-antagonists monotherapy was also found to have higher reported hospitalization rates (14% *vs*. 8%) (34).

Strengths of the present study include its prospective design, which facilitated the collection of a comprehensively characterized clinical dataset, all of which was registered at time of sampling. In keeping with this, we employed a predetermined time interval for blood sampling in order to measure anti-SARS-CoV-2 antibody concentrations at comparable intervals after the last vaccination that patients received. For anti-SARS-CoV-2 antibody detection, we leveraged a high-sensitive, specific, and validated electro chemiluminescent microparticle immunoassay (CMIA), of which test characteristics have been associated with those from neutralizing assays that are generally considered to be more biologically appropriate (36, 37).

There are several limitations to our study that warrant recognition. First, our study was of cross-sectional origin, in the absence of longitudinal data or a prolonged follow-up, which precluded the assessment of anti-SARS-CoV-2 antibody concentrations at later stages. Furthermore, included subjects were not randomized based on drug treatment regimens, thus confounding by indication could not be fully excluded, resulting in varying distributions of subjects among the types of medications analyzed. Second, we only quantified antibodies against the receptor-binding domain (RBD) of the SARS-CoV-2 spike protein and thereby assessed the humoral immune response, whereas we did not focus on cellular immunity or (memory) T-cell responses after full vaccination. Anti-SARS-CoV-2 spike (S) antibody concentrations correlate with the protection against disease severity of SARS-CoV-2 (38, 39). However, multiple studies fairly point to the fact that T-cell-mediated immunity, independent of antibody formation, is equally essential for achieving an adequate immunological protection (39, 40). Third, objective evidence for prior SARS-CoV-2 infections was lacking, as a history of COVID-19 was solely patient-reported. Since SARS-CoV-2 PCR testing facilities have been insufficient in the Netherlands in the first months of the COVID-19 pandemic, possible prior SARS-CoV-2 infections could have been missed for selected patients.

In conclusion, we show that the use of TNF-α-antagonists in patients with IBD is strongly associated with an attenuated serological response after full vaccination against SARS-CoV-2, which was independent of the elapsed time since last vaccination and blood sampling, prior SARS-CoV-2 infection, and patient age. Based on our data, we do not advocate to pre-emptively discontinue any type of medical treatment for IBD as seroconversion rates are generally very high after full vaccination (98.3% in our cohort), despite the use of immunomodulating treatment. Furthermore, the concentration of anti-SARS-CoV-2 antibodies does not necessarily correlate with the level of protection against novel infection. Therefore, we fully support consensus-based recommendations stating that patients with IBD should preferably be fully vaccinated against SARS-CoV-2, in particular with mRNA-based vaccines, that specific subgroups of patients may benefit from booster doses (vaccine prioritization).

## Data Availability Statement

The raw data supporting the conclusions of this article will be made available by the authors, without undue reservation.

## Ethics Statement

The studies involving human participants were reviewed and approved by Medisch Ethische Toetsingscommissie (METc), UMCG. The patients/participants provided their written informed consent to participate in this study.

## Author Contributions

All authors were involved in conceptualization and study design. GD and MV were responsible for funding acquisition and resources. MV provided supervision. AO, AB, PH, HM, and MV collected study data and materials. AB and AO performed data curation and data analysis. AB performed data visualization. AO and AB wrote the first draft of the manuscript. All authors contributed to results interpretation, critically reviewed the manuscript, contributed to manuscript revision, and read and approved the final version of the manuscript.

## Funding

The research position of AB was supported by a JSM M.D.-Ph.D. trajectory grant from the Junior Scientific Masterclass (JSM) of the University of Groningen, the Netherlands (no: 17-57). The funders had no role in the design of the study, collection, analysis, or interpretation of data, nor in writing of the manuscript.

## Conflict of Interest

GD received research grants from Royal DSM and speaker’s fees from Janssen Pharmaceuticals, Takeda, Pfizer and Abbvie. RW acted as consultant for Takeda, received unrestricted research grants from Takeda, Johnson & Johnson, Tramedico and Ferring, and received speaker fees from MSD, Abbvie and Janssen Pharmaceuticals. MV has served on the advisory board for Janssen-Cilag and received a speaker’s fee from Takeda, outside the submitted work. The funders had no role in the design of the study, collection, analysis, or interpretation of data or in writing the manuscript.

The remaining authors declare that the research was conducted in the absence of any commercial or financial relationships that could be construed as a potential conflict of interest.

## Publisher’s Note

All claims expressed in this article are solely those of the authors and do not necessarily represent those of their affiliated organizations, or those of the publisher, the editors and the reviewers. Any product that may be evaluated in this article, or claim that may be made by its manufacturer, is not guaranteed or endorsed by the publisher.
